# Eriocalyxin B Inhibits Adipogenesis in 3T3-L1 Adipocytes by Cell Cycle Arrest

**DOI:** 10.1007/s13659-020-00240-6

**Published:** 2020-04-20

**Authors:** Rong-Fang Mu, Yan-Fen Niu, Qian Wang, Hui-Min Zhou, Jing Hu, Wan-Ying Qin, Wen-Yong Xiong

**Affiliations:** 1 State Key Laboratory of Phytochemistry and Plant Resources in West China, Kunming Institute of Botany, Chinese Academy of Sciences, Kunming, 650201 Yunnan People’s Republic of China; 2 Key Laboratory of Medicinal Chemistry for Natural Resource, Yunnan University, Kunming, 650500 Yunnan People’s Republic of China; 3 Biomedical Engineering Research Center, Kunming Medical University, Kunming, 650500 Yunnan People’s Republic of China; 4 University of Chinese Academy of Sciences, Beijing, 100049 People’s Republic of China

**Keywords:** Eriocalyxin B, Adipocyte differentiation, Cell cycle

## Abstract

**Abstract:**

Eriocalyxin B, an ent-Kaurene diterpenoid extracted from a traditional Chinese herb *Isodon eriocalyx*, has been shown to possess multifunctional activities such as anti-cancer and anti-inflammatory. However, the function and mechanism of the compound in adipocyte differentiation is still unknown. Here we reported that eriocalyxin B blunted adipogenesis remarkably by inhibiting the accumulation of lipid droplets, triglycerides and the expressions of adipogenesis-related factors, including C/EBPβ, C/EBPα, PPARγ, and FABP4. Moreover, we showed that the inhibition might be the consequence of cell cycle being arrested at the G2/M phase during the mitotic clonal expansion of adipocyte differentiation, most likely by suppressing mRNAs and proteins of CDK1, CDK2, Cyclin A and Cyclin B1. Overall, we conclude that eriocalyxin B is capable of inhibiting adipocyte differentiation at the early stage through downregulating the proteins involved in cell cycle progression.

**Graphic Abstract:**

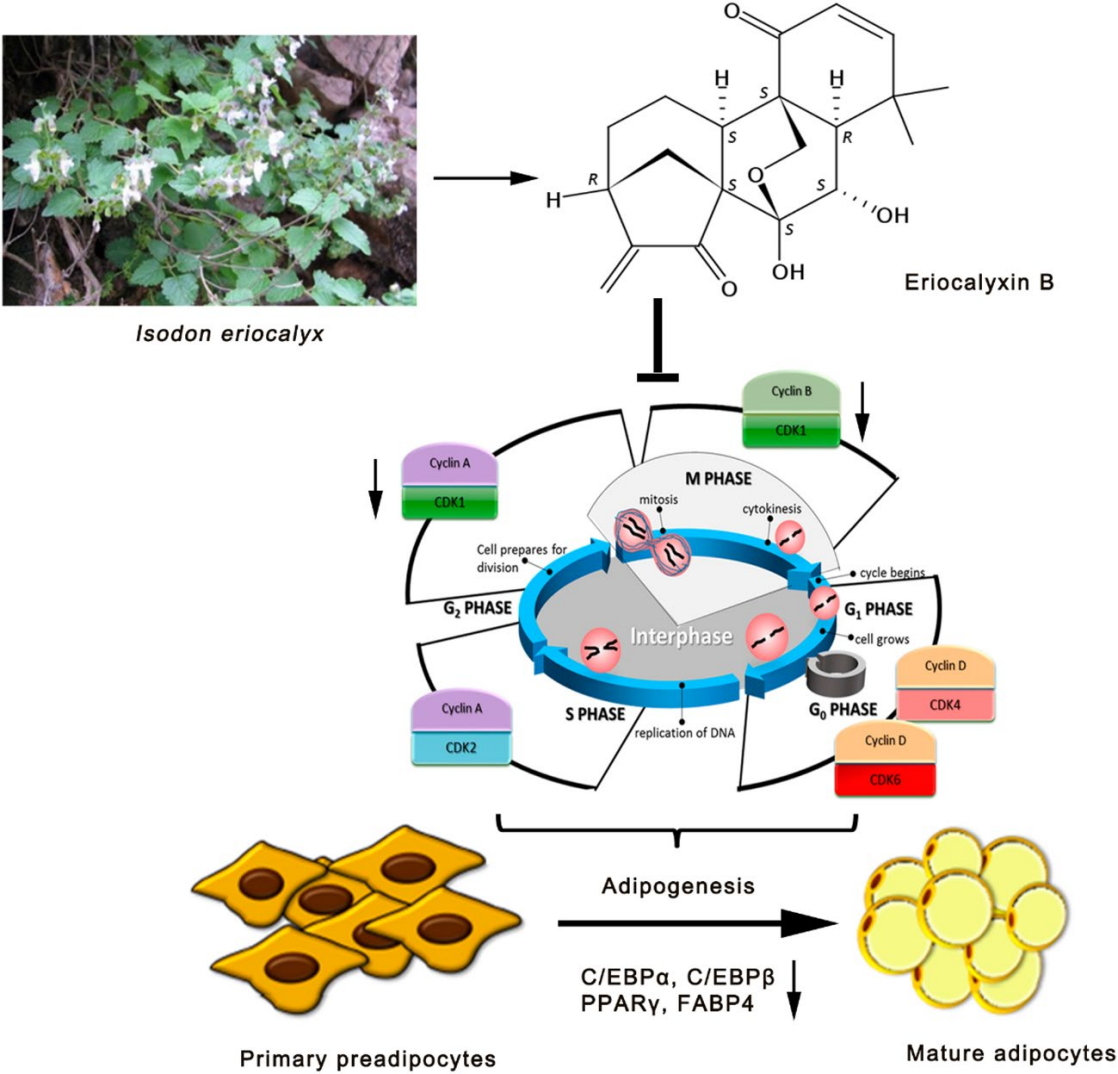

## Introduction

With the rapid development of modern society, the epidemic of overweight and obesity has brought huge medical burden to our society. According to the World Health Organization, obesity is not only a physical trait but also a chronic disease, which is regarded as a high-risk factor for various diseases including diabetes, non-alcoholic fatty liver disease, cardiovascular disease, hypertension and cancers [1–4]. Obesity is a disorder which is accompanied to the unbalance of energy intake and expenditure [5, 6]. As we are known that obesity is characterized by the increased size (hypertrophy) and number (hyperplasia) of adipocytes in adipose tissue, which results in excessive lipid accumulation in white adipose tissue [7, 8].

In theory, reducing calorie intake and increasing expenditure are the methods for overcome obesity. In addition, other approaches are used for the treatment of obesity, such as surgical treatment and medication. So far, the US FDA has approved six major therapeutic drugs for obesity treatment: phentermine, orlistat, lorcaserin, liraglutide, naltrexone/bupropion sustained release, and phentermine/topiramate extended release [9]. However, most of anti-obesity medications were withdrawn from the market due to their useless effects or undesirable side effects. Examples of adverse effects include diarrhea, dry mouth, dyspepsia (orlistat), pulmonary hypertension (aminorex), stroke (phenylpropanolamine), and neuropsychiatric issues (rimonabant) [10, 11]. These undesirable experiences highlight the importance of safety assessments and necessitate the development of new drug development strategy to achieve more efficient and safer obesity treatment options. Traditional medicinal plants have huge natural compound resources and better safety in drug development and application. At present, several studies revealed the effective natural compounds of antiobesity are extracted from botany, including sulforaphene [12], daidzein [13], quercetin [14], hesperidin and capsaicin [15], methylated cereal flavonoid [16], and dietary phenolic [17], indicating the natural products are a great choice as potential candidates for treatment of obesity.

In vitro, the 3T3-L1 cell line is considered as a well-established and classic cell line that was frequently used for study of adipocyte differentiation, lipid metabolism, insulin signaling, regarding of its differentiation process involving both hypertrophy and hyperplasia of obesity [18, 19]. The adipocyte differentiation is regulated by several adipogenic-specific genes, such as peroxisome proliferator-activated receptorγ (PPARγ), CCAA/enhancer-binding protein (C/EBP) family members and fatty acid binding protein (FABP) [20–23]. Moreover, the adipocyte differentiation is extensively regulated through crosstalk between the cell cycle regulators and adipogenic transcription factors [12, 24, 25]. The process of adipogenesis is divided into several phases, including growth arrest, mitotic clonal expansion (MCE), lipid accumulation, and late phase of differentiation [26]. Especially, MCE is an essential stage for terminal differentiation, which results in an increase in cell numbers [27]. Additionally, a number of proteins participated in MCE, the progression of cell cycle are largely regulated by the activation of cyclin-dependent kinases (CDKs), and cyclins which are considered as critical determinants for early adipocyte differentiation program [28]. Many studies show that MCE is a prerequisite for differentiation of 3T3-L1 preadipocytes into adipocytes. A study demonstrated that Eriocalyxin B markedly arrested cell cycle at G2/M phase in a dose-dependent manner [29]. CDKs are serine/threonine kinases that control cell cycle progression. CDKs are activated by cyclins and act as a regulatory subunit. These CDK/cyclin complexes are fundamental to the orderly progression of the cell [30].

Eriocalyxin B, isolated and identified in 1982 by Han-dong Sun’s group, is the major component in Chinese plant *Isodon eriocalyx* (Dunn.) Hara (family Lamiaceae) showing many pharmacological activities, such as inhibiting inflammatory response, regulating immune cell differentiation, inhibiting tumor cells proliferation, causing cell cycle arrest affecting angiogenesis and promoting cancer cells apoptosis [31–33]. However, it is unknown whether Eriocalyxin B functions in adipocyte differentiation. Therefore, in this work we aimed to elucidate whether Eriocalyxin B could prevent adipogenesis and address the potential mechanism.

## Results

### Eriocalyxin B Inhibited Adipocyte Differentiation

**Fig. 1:**
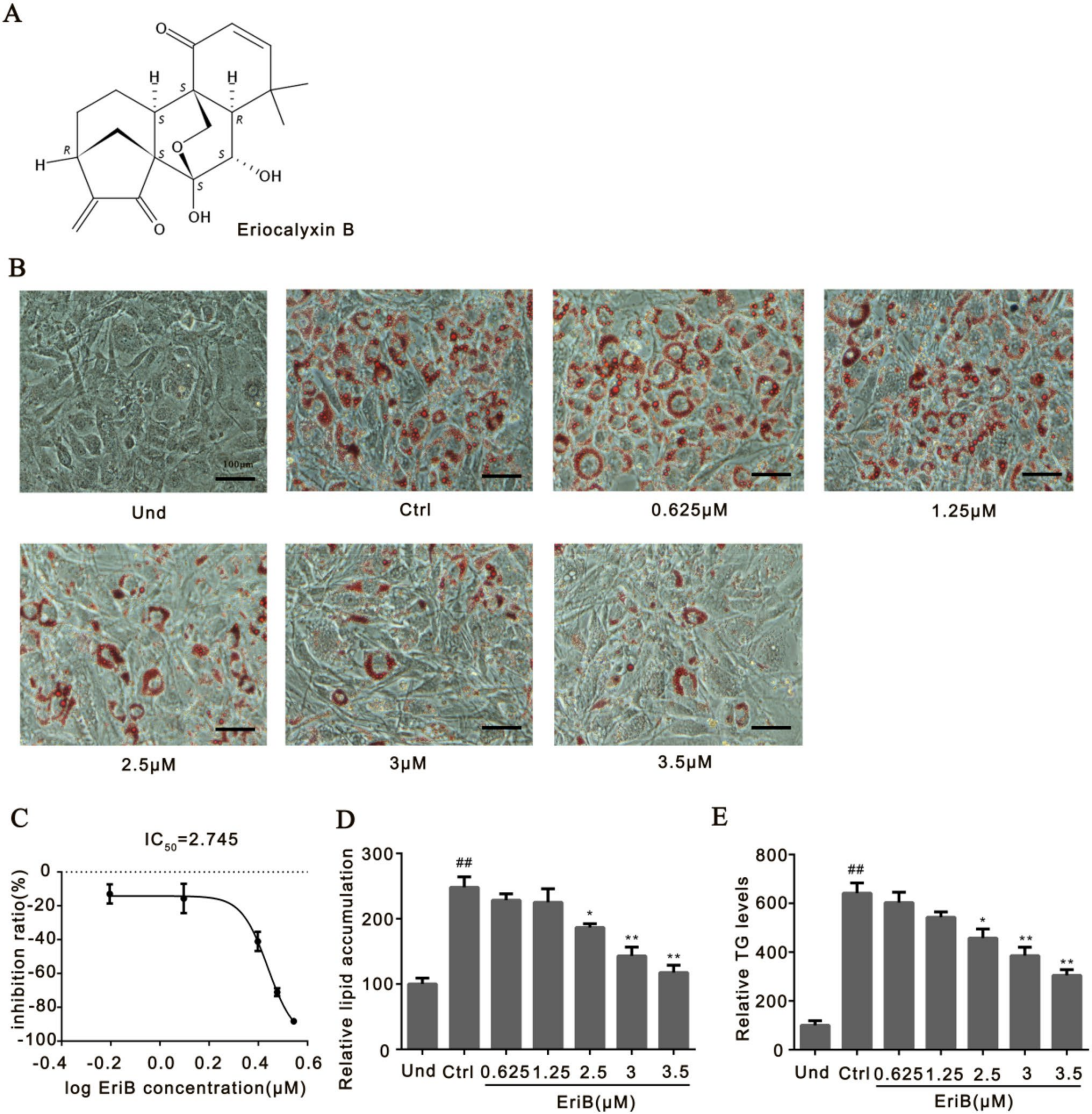
Eriocalyxin B inhibited adipogenesis. **a** Chemical structure of eriocalyxin B. 3T3-L1 preadipocytes were incubated with indicated concentrations of eriocalyxin B for 7 days. **b** Representative images of 3T3-L1 cells treated with the inducing cocktail and a series of concentrations of eriocalyxin B and stained with Oil red O. Scale bar, 100 μm. **c** Percentage of inhibition of the eriocalyxin B on adipogenesis of **b**. **d** Normalized lipid accumulation of the cells from **b**. **e** Normalized TG levels of the cells from **b**. The Data were presented as mean ± SEM (##*p* < 0.01 compared to the undifferentiated group, Und; *p < 0.05, **p < 0.01 compared to Ctrl) from three independent experiments

The 3T3-L1 cells, a classic cell model for adipogenesis in vitro, were treated with a widely-used adipogenesis inducing cocktail for 7-day [34]. At the end of adipogenesis induction, the level of lipid accumulation in adipocytes was stained by Oil Red O and followed by OD measurements. As shown, the lipid accumulation of the cells were fully differentiated after inducing (Ctrl), whereas the accumulations were blunted by the treatment of Eriocalyxin B (Fig. 1b), which were quantified by the OD values of Oil Red O from the cells (Fig. 1c–d). The half inhibitory concentration of Eriocalyxin B for blunting adipogenesis is about 2.745 μM (Fig. 1d). These inhibiting results were insured by measuring the TG contents in these cells treated by the compound (Fig. 1e), supporting that the compound blunted adipogenesis with a dose-dependent manner.

### Eriocalyxin B Suppressed Key Regulators in Adipocyte Differentiation

**Fig. 2:**
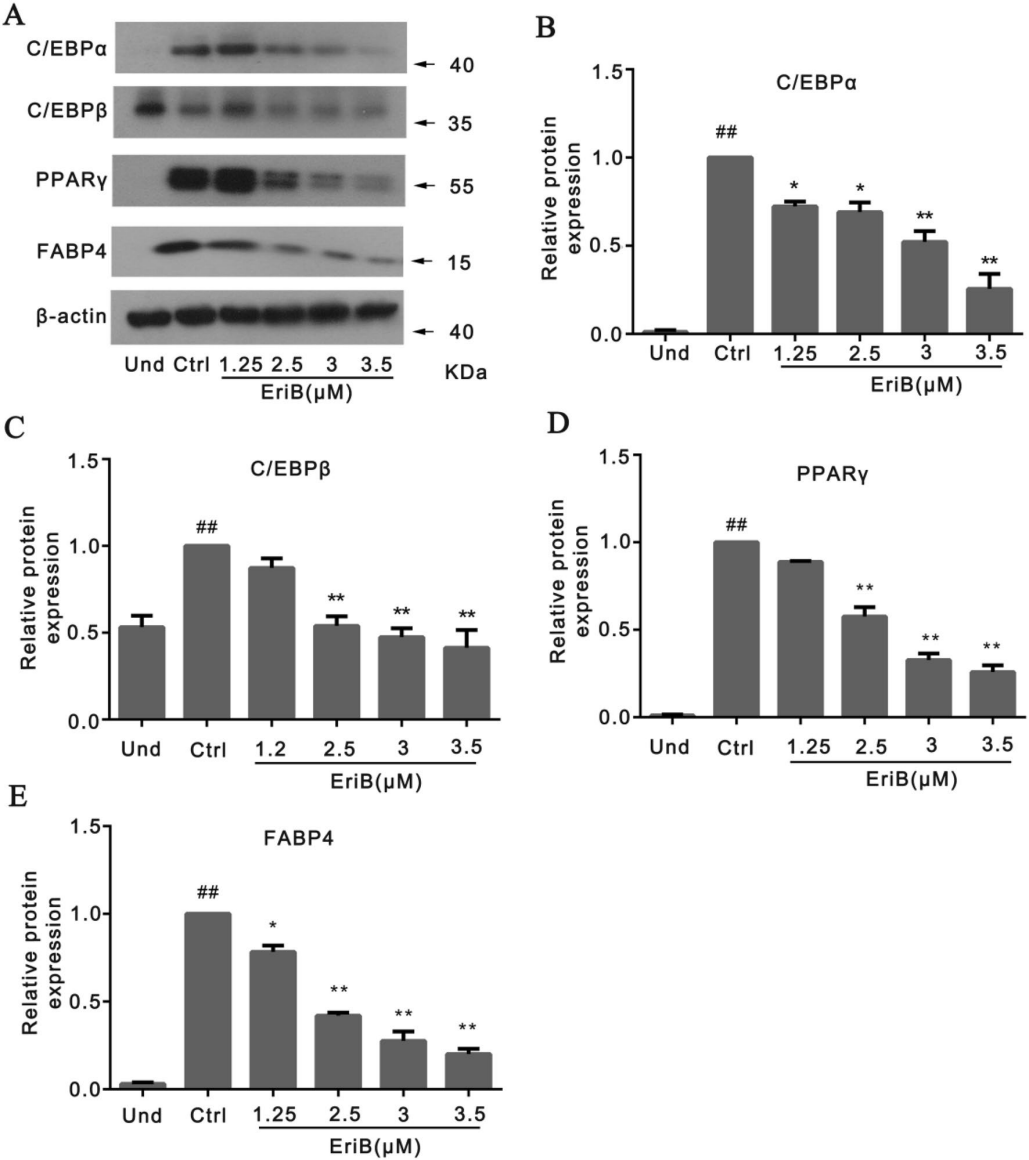
Eriocalyxin B blunted the key regulators in adipocyte differentiation. **a** Western blotting of the cells treated with a series of doses of eriocalyxin B as indicated for C/EBPα, C/EBPβ, PPARγ and FABP4. **b**–**e** Quantified and normalized protein levels of C/EBPα, C/EBPβ, PPARγ and FABP4 respectively from **a**. Data were presented as mean ± SEM (## *p* < 0.01 compared to Und, *p < 0.05, **p < 0.01 compared to Ctrl) from three independent experiments

Since we have demonstrated the effect of eriocalyxin B in adipogenesis above, we next detected the expressions of several key regulator in adipogenesis, including C/EBPα, C/EBPβ, PPARγ and FABP4 [20–23]. As expected, the regulators were all upregulated at the end of differentiation (Ctrl group), whereas these regulators were all gradually downregulated following the gradually increases of the compound’s concentrations (Fig. 2a). The values were further quantified as following: C/EBPα (Fig. 2b), C/EBPβ (Fig. 2c), PPARγ (Fig. 2d) and FABP4 (Fig. 2e), supporting the effect of the compound in blunting the process of adipogenesis (Fig. 1).

### Eriocalyxin B Blunted the Adipogenesis at the Early Stage

**Fig. 3:**
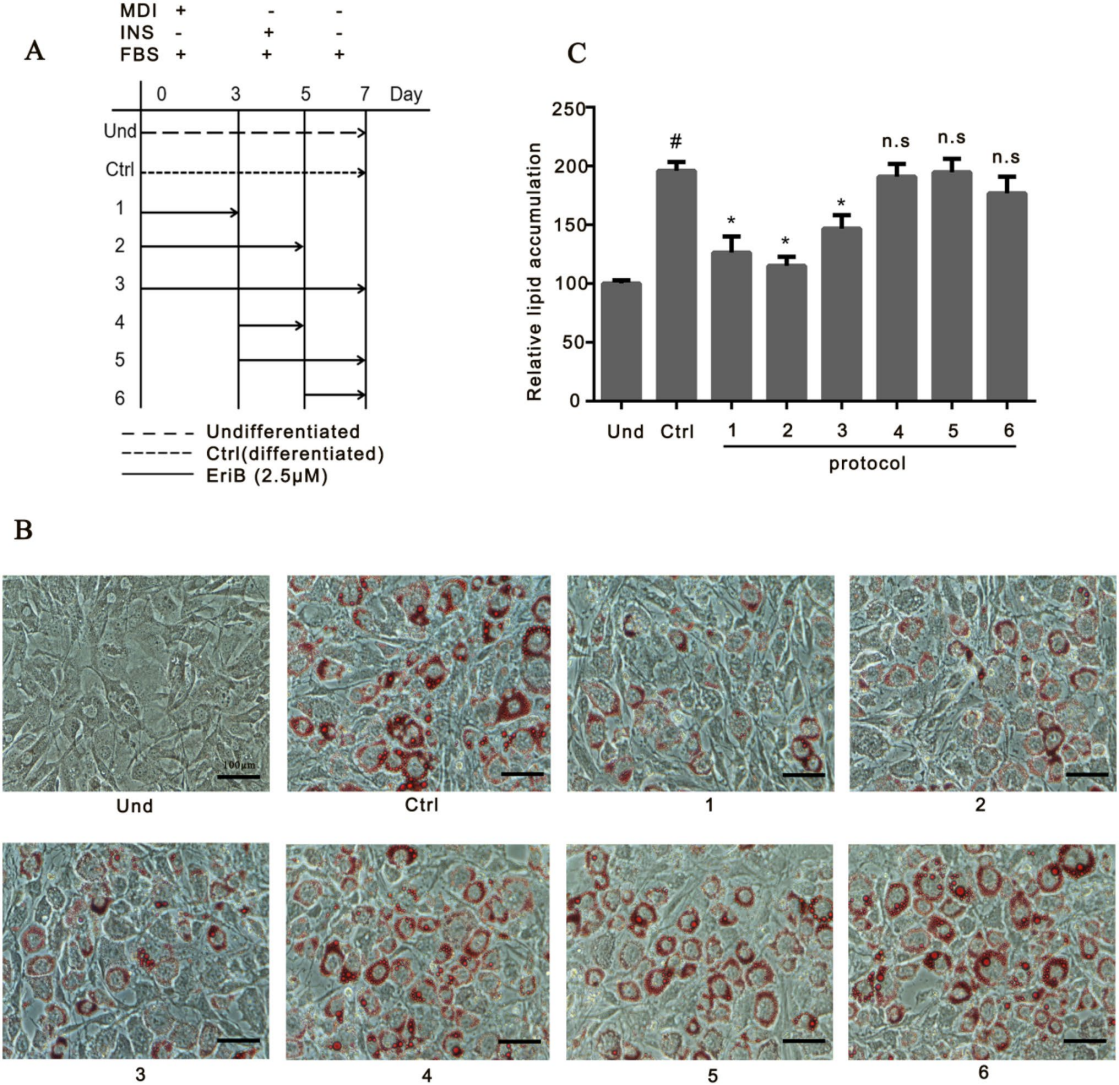
Eriocalyxin B inhibited adipogenesis at the early stages of adipocyte differentiation. Cells were induced for differentiation with DMEM supplemented with 10% FBS, IBMX, DEX and insulin (described as MDI). On day 3, the medium was changed to medium containing 10% FBS and insulin for 2 days (called as INS). On day 5, insulin was removed from 10% FBS-DMEM for another 2 days (described as FBS). The cells were fully differentiated into mature adipocytes on day 7. Cells treated with of compound at different times. **a** Schematic diagram of the protocols for cells treated with eriocalyxin B during the 7-day process of inducing of differentiation. **b** Representative images of the cells treated as protocol in A and stained by Oil Red O. **c** Quantified and normalized lipid accumulation in the cells from **b**. Data were presented as mean ± SEM (#*p* < 0.05 compared to Und; *p < 0.05 compared to Ctrl; n.s., compared to Ctrl) from three independent experiments

Next we aimed to investigate how eriocalyxin B affect differentiation of adipocytes. Here we treated 3T3-L1 cells with eriocalyxin B (2.5 μM) at the different stages of the 7-day induction of adipogenesis (Fig. 3a). Our data showed that adding eriocalyxin B to the induction mix during 0–3 days, 0–6 days and 0–7 days (protocol 1, 2, 3 respectively) of the adipogenesis process resulted in the blunting of adipogenesis significantly, whereas the adding to the inducing mix after the 3rd (protocol 4–6) day of induction did not inhibited adipogenesis significantly, suggesting that the compound functions in adipogenesis in the early stage of adipocyte differentiation.

### Eriocalyxin B Arrested Cell Cycle Progression During the MCE Period of Adipogenesis

During the earlier process of adipogenesis, the growth-arrested 3T3-L1 preadipocytes go through two sequential rounds of mitosis in the initial 48 h of differentiation which is termed as MCE. Above result has shown that Eriocalyxin B functioned in the early phase, including MCE of differentiation, therefore we perform cell cycle assay to address whether Eriocalyxin B affects the cell cycle progression during MCE.

**Fig. 4:**
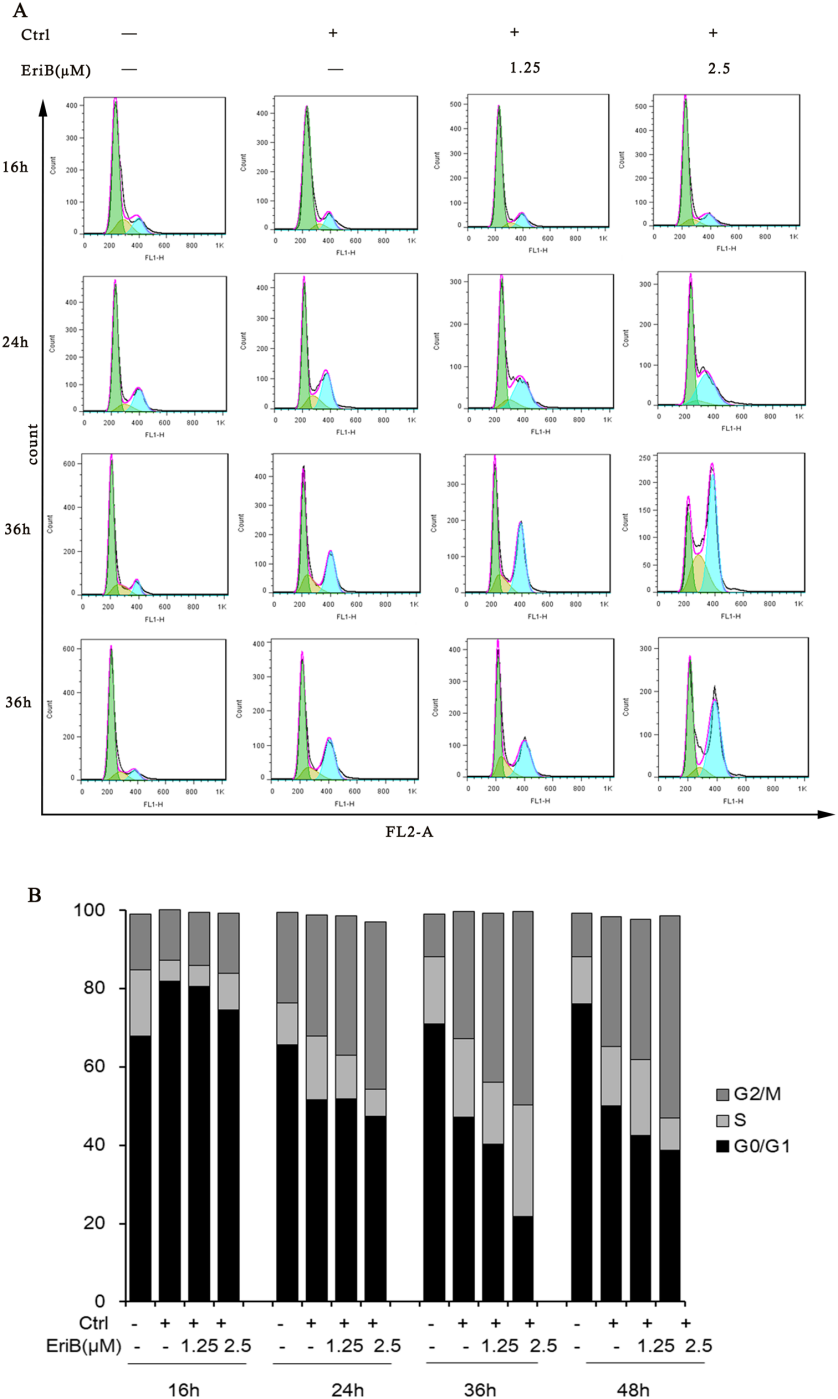
Eriocalyxin B induced cell cycle arrest at G2/M checkpoint of the MCE period during adipocyte differentiation. **a** Flow cytometry analysis of cells treated with a series of doses of eriocalyxin B for 16, 24, 36 or 48 h. **b** Quantification of the cells from **a**. Data were averages of three independent experiments

As expected, by flow cytometric analysis, the undifferentiated cells did not undergo cell cycle progression because most of cells (~ 65%) resided in G0/G1 phase from 16 to 48 h, whereas in the differentiated group (Ctrl), the percentage of cells in G2/M phase were 12.99%, 30.84%, 32.41% and 33.13% at four time points of 16, 24, 36 and 48 h (Fig. 4). Interestingly, when cells were treated with 2.5 μM Eriocalyxin B, the percentage of G2/M phase cells was 15.31%, 42.8%, 49.34%, and 51.74% respectively. The results displayed that the cells aggregated in the G2/M phase is compared with the Ctrl group, supporting that eriocalyxin B blocks the cell cycle progression by arresting the cells in G2/M phase during the MCE period of adipogenesis.

### Eriocalyxin B Suppressed the Expressions of the Regulators for Cell Cycle

**Fig. 5:**
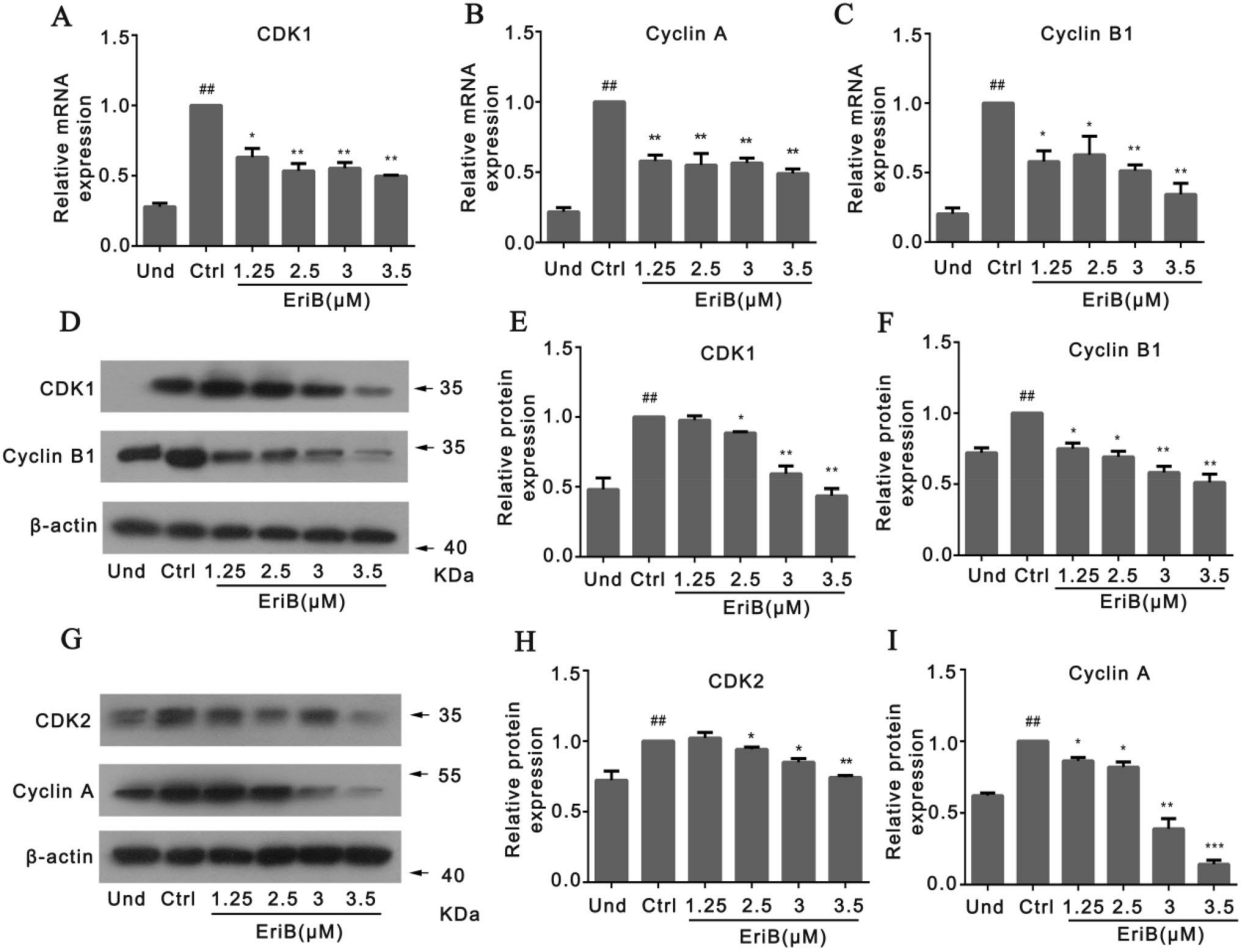
Eriocalyxin B down-regulated the regulators for cells cycle. **a-c** The mRNA levels of CDK1, Cyclin A and Cyclin B1 of the cells treated by 2.5 μM eriocalyxin B for 24 h. **d** Western blotting of CDK1 and Cyclin B1 of the cells treated by eriocalyxin B for 24 h. **e**–**f** Normalized protein levels of samples in **d** for CDK1 and Cyclin B1 respectively. **g** Western blotting of CDK2 and Cyclin A of the cells treated by eriocalyxin B. **h**–**i** Normalized protein levels of samples in G for CDK2 and Cyclin A respectively. The data were presented as mean ± SEM (##*p* < 0.01 compared to Und, *p < 0.05, **p < 0.01, ***p < 0.001 compared to Ctrl) from three independent experiments

To further investigate the mechanism of the eriocalyxin B on the cell cycle arrest, we assessed the key cell cycle regulators in the G2/M phase, including CDK1, CDK2, Cyclin A, and Cyclin B1. The mRNA levels of CDK1, Cyclin A and Cyclin B1 were suppressed by eriocalyxin B as determined at 24 h after differentiation (Fig. 5a–c), which is consistent with result of cell cycle (Fig. 4). Moreover, in parallel with the result of mRNA levels of these regulators, the protein levels of CDK1, CDK2, Cyclin A and Cyclin B1 were also down-regulated by eriocalyxin B treatment (Fig. 5d–i), confirming the eriocalyxin B inhibited the cell cycle progression during MCE period of adipogenesis.

## Discussion

Eriocalyxin B has been documented to possess a variety of pharmacological activities, which has the effects of anti-inflammatory, antiproliferative, anti-angiogenic, and the most common activity is anti-cancer [29, 35, 36]. In this work, we reported that eriocalyxin B is capable of inhibiting adipocyte differentiation.

Experimental data has shown that eriocalyxin B exerts biological properties through arresting cell cycle, prompting apoptosis, and modulating cell signaling pathway, which modulates multiple cell signaling pathways including NF-κB, MAPK, JAK, STAT3, NOTCH, and Wnt pathways [36–41]. Eriocalyxin B prevented the cancer cells proliferation by inhibiting the progression of cell cycle, which affected cell cycle related regulators, including CDK, Cyclins and CKI [31, 41]. Our study further explored the molecular mechanism and found that eriocalyxin B suppressed adipocyte differentiation by causing cell cycle arrest in 3T3-L1 adipocytes. The 3T3-L1 cells we use are normal tissue cells (adipocytes) rather than cancer cells. Therefore, we speculate that the inhibitory effect of eriocalyxin B on adipogenesis may be a benign “side effect” when it exerts anti-cancer function. Of course, this inference needs to be fully addressed in the future.

In conclusion, eriocalyxin B, a natural entkaurene diterpene compound isolated from *Isodon eriocalyx*, is able to inhibit the early stage of adipogenesis by suppressing the signaling proteins involved in cell cycle progression.

### Experimental Section

#### Chemicals and Reagents

Adipocyte growth medium (DMEM) were purchased from Biological Industries (Israel). The fetal bovine serum (FBS) for cell culture was purchased from GIBCO BRL (Grand Island, NY, USA). Penicillin/streptomycin (P/S) and Calf serum (CS) were obtained from Biological Industries (Israel). Rosiglitazone (Rosi), dimethylsulfoxide (DMSO) and 3-isobutyl-1-methylxanthine (IBMX) were obtained from Sigma-Aldrich (St Louis, MO, USA). Insulin was from Roche (Switzerland), and dexamethasone (DEX) was from Adamas (Switzerland). Bovine serum albumin (BSA) and TRIzol reagent were from Shanghai Sangon Biotech (Shanghai, China). QuantiSpeed SYBR kit was from novopretion (China). Triglycerides Kit was purchased from Nanjing Jiancheng Bioengineering Institute. The primary antibodies against β-actin, FABP4, C/EBPβ, CDK2, C/EBPα and PPARγ were from Cell Signaling Technology (Beverly, MA, USA) and CDK1, Cyclin A, Cyclin B1 from Abcam (USA) and HuaBio (China).

#### Cell Culture and Differentiation

The 3T3-L1 murine pre-adipocytes cell line was purchased from the American Type Culture Collection (ATCC, Manassas, VA, USA). Cells were routinely cultured in DMEM supplemented with 10% CS and 1% P/S at 37 ℃ in a 5% CO_2_ atmosphere. Two days after confluence (day 0), cells were induced for differentiation with DMEM supplemented with 10% FBS, IBMX, DEX and insulin (MDI) as previously described (Jing Hu, Molecules 2019). On day 3, the medium was changed to medium containing 10% FBS and 1 μg/mL insulin for 2 days (day 5), and then insulin was removed from 10% FBS-DMEM for another 2 days. The cells were fully differentiated into mature adipocytes on day 7.

#### Oil Red O Staining

After removing the culture medium, 3T3-L1 cells were washed three times with PBS and subsequently fixed in 10% formaldehyde for 1 h at room temperature. After fixing, the cells were washed with water twice and one time with 60% isopropanol then the cells were stained with Oil Red O working solution for 30 min at room temperature, washed with water three times, and then photos were taken under microscopy. To quantify the lipid accumulation, the stained 3T3-L1 cells were washed with 100% isopropanol and the absorbance was measured at 492 nm by using a microplate reader (Perkin Elmer Envision Multilabel reader).

#### Measurement of Intracellular Triglyceride

After treatment with eriocalyxin B, rinsed cells with 100% isopropanol. The intracellular lipid was collected by isopropanol to detect triglycerides (Triglycerides Kit, Biosino Bio-Technology and Science Incorporation, China).

#### Cell Cycle Assay

3T3-L1 cells were induced to differentiate in DMI medium with or without eriocalyxin B for 16 h, 24 h, 36 h and 48 h. The cells were then harvested, washed with PBS, fixed in 70% pre-cooling ethanol overnight at − 20 °C, washed with PBS, resuspended in 500μL of PBS containing 100 μg/mL RNase for 30 min at 37 °C and subsequently incubated with the nuclear stain PI at a final concentration of 50 μg/mL for 15 min at 37 °C. The stained cells were analyzed by using a BD AccuriC6 flow cytometer (BD Biosciences, San Jose, CA, USA) and the cell cycle distribution was analyzed by using FlowJo software.

#### RNA Isolation and Real-time PCR

RNA was extracted from 3T3-L1 cells using TRIzol reagent (TIANGEN BIOTECH), and cDNA was synthesized using cDNA synthesis kits using total RNA (2 μg) (Applied Biological Materials Inc). Real-time PCR cDNA gene expression was detected using the SYBR Green Master kit and a spectrofluorometric thermal cycler (Applied Biosystems). *β-actin* was used as a housekeeping gene to evaluate the relative expression of genes.

**Table:** 

Gene	Forward primer	Reverse primer
*CDK1*	AAGCCGCTTTTCCACGGC	GCTCCCCGGCTTCCACTT
*CyclinA*	ACCTCAAAGCGCCACAACAT	CAAAGCCGGCAGTCTTTCAC
*CyclinB*	GCTGGTCGGTGTAACGGC	GGCGACCCAGGCTGAAGT
*β-actin*	CACCCCAGCCATGTACGT	GTCCAGACGCAGGATGGC

### Western Blotting

Western blot analysis was performed for the expression of adipogenesis-associated proteins. Cells were lysed in RIPA extraction buffer (Beyotime, Haimen, China) on ice for 30 min. The proteins were subjected to 10% SDSPAGE and transferred to a PVDF membrane (Merck Millipore, Billerica, MA, USA) for 90 min. After blocking with 5% skim milk, the membrane was incubated with a primary antibody at 4 °C overnight using the indicated commercial antibodies (PPARγ, FABP4, CEBPα, CEBPβ, CDK1, CDK2, Cyclin A, Cyclin B1, *β*-actin). Subsequently, the membranes were incubated with secondary antibodies and then developed using Western Lightning Chemilum-inescence Reagent (Perkin-Elmer Life Science, MA, USA). Finally, the immunoblots were quantified using the Metamorph software.

### Statistical Analysis

All data are presented as the means ± SEM of triplicate experiments. An analysis of variance (ANOVA) and Student’s t-test were used to determine the significance of difference means. *P* values less than 0.05 were considered to be statistically significant.
