# Correction to: Eriocalyxin B Inhibits Adipogenesis in 3T3‑L1 Adipocytes by Cell Cycle Arrest

**DOI:** 10.1007/s13659-020-00251-3

**Published:** 2020-06-16

**Authors:** Rong‑Fang Mu, Yan‑Fen Niu, Qian Wang, Hui‑Min Zhou, Jing Hu, Wan‑Ying Qin, Wen‑Yong Xiong

**Affiliations:** 1 State Key Laboratory of Phytochemistry and Plant Resources in West China, Kunming Institute of Botany, Chinese Academy of Sciences, Kunming, 650201 Yunnan People’s Republic of China; 2 Key Laboratory of Medicinal Chemistry for Natural Resource, Yunnan University, Kunming, 650500 Yunnan People’s Republic of China; 3 Biomedical Engineering Research Center, Kunming Medical University, Kunming, 650500 Yunnan People’s Republic of China; 4 University of Chinese Academy of Sciences, Beijing, 100049 People’s Republic of China

## Correction to: Natural Products and Bioprospecting (2020) 10:131–140 10.1007/s13659-020-00240-6

In the original publication of this article, we found an error under the section “Introduction”. The first sentence of the fourth paragraph appears incorrectly. The corrected sentence is given below.

Eriocalyxin B, isolated and identified in 1982 [1], is the major component in Chinese plant *Isodon eriocalyx* (Dunn.) Hara (family Lamiaceae) showing many pharmacological activities, such as inhibiting inflammatory response, regulating immune cell differentiation, inhibiting tumor cells proliferation, causing cell cycle arrest affecting angiogenesis and promoting cancer cells apoptosis.
